# Meaning making in retirement transition: a qualitative inquiry into Slovak retirees

**DOI:** 10.1080/17482631.2021.1985414

**Published:** 2021-10-25

**Authors:** Peter Halama, Lucia Záhorcová, Žaneta Škrobáková

**Affiliations:** a Institute of Experimental Psychology, Centre of Social and Psychological Sciences, Slovak Academy of Sciences, Bratislava, Slovakia; b Institute of Applied Psychology, Faculty of Social and Economic Sciences, Comenius University, Bratislava, Slovakia

**Keywords:** Meaning making, retirement, qualitative analysis

## Abstract

**Purpose:** This study is a qualitative inquiry into meaning making during retirement transition. The study focuses on how Slovak retirees reconstruct meanings during the transition and the factors which both help and hinder this process.

**Methods:** Forty individuals (M = 63.36; SD = 2.47) who had recently transitioned into retirement were interviewed and data were analysed using the Consensual Qualitative Research-Modified approach.

**Results:** The analysis generated five basic domains with categories and subcategories of the participants’ responses. The analysis showed that once retired, the participants generally continued to rely on previous meaning sources such as work and family; however, there were changes such as switching from job-related work to work related to hobbies and housekeeping, or from financially providing for the family to maintaining family relationships and grandparenting. The main factors facilitating the meaning making process were positive attitudes and social support for meaning. The risk factors included lack of finances, poor health of retiree or a close person, and the loss of a spouse.

**Conclusions:** In general, the research showed that the main features of the retirees’ meaning making processes were maintaining accessible sources, compensating for sources lost during the transition, and managing beneficial and risk factors.

Retirement belongs to the most important life transitions that can affect personal resources and well-being (Gall et al., 1997; Osborne, 2012). During the adjustment process, people try to adapt to their retirement-related life changes by rebuilding their life in different areas—financially, socially, emotionally, or motivationally (Martinčeková & Škrobáková, 2019; Wang et al., 2011; Záhorcová et al., 2021). Although dynamic changes in Western society associated with increasing life expectancy and a decreasing birth rate have changed society’s view of retirement and enabled a more flexible and continual disengagement from work, a diminishing psychological commitment to work and behavioural withdrawal from work are still key components of the retirement process (Shulzt & Wang, 2011).

Managing retirement involves two major developmental challenges: social and psychological detachment from work and developing a satisfactory post-retirement lifestyle (Van Solinge & Henkens, 2008). Successfully resolving these challenges contributes to good adaptation to retirement and overall psychological well-being and satisfaction in retirement. In previous research, several models of retirement adaptation have been adopted to explain how people adapt to retirements and what makes this process successful, such as role theory (Barnes-Farrell, 2003), continuity theory (Atchley, 1989), and life course perspective theory (Wang & Shultz, 2010). An example of an integrative and empirically testable model is one proposed by Wang et al. (2011): the resource-based dynamic model for retirement adjustment. It is based on the assumption that good adjustment is the direct result of the individual’s access to resources. This means that people with more resources to fulfil their needs in retirement will adjust better to retirement. On the contrary, people with diminishing resources after retirement will experience difficulties in adjusting. Therefore to understand retirement adjustment, researchers should examine variables with a direct impact on retirees’ resources.

The improvements in quality of life and healthcare of the past decades have significantly changed the retirement process. People frequently retire in relatively good health, with several years of life still ahead of them (Malette & Oliver, 2006) and this brings a new kind of challenge—the need to find new purpose and meaning in the next stage of life. Retirement can sometimes mean a person has limited resources for deriving meaning in life. Employment and work can be a source of meaning in many ways (Steger & Dik, 2010). They provide a source of income, a life routine structuring the use of time, they may be a source of personal identity and social interaction, and provide a meaningful experience and sense of accomplishment (Moen, 1996). Withdrawal from work substantially reduces these sources and newly retired people face the challenge of coping with these reductions. Previous research has shown that older people are at greater risk of life losing meaning for them than younger people (Halama, 2007; Pinquart, 2002; Reker et al., 1987), although some studies have indicated the opposite (Steger et al., 2009). Pinquart (2002) reasoned that older people may have increasing difficulty finding meaning in life as age-associated disruptions in social and psychological engagement are likely to deprive them of important sources of purpose in life. These disruptions may relate to retirement as well as to other age-related situations, such as the loss of a close person or illness. Other reasons for meaning to fade in old age are the lack of clearly defined roles, norms, and opportunities for older adults (Pinquart, 2002; Ryff & Singer, 1998). A weaker sense of meaning in life and other retirement-related changes can lead to retirement being perceived as a stressful life event (Holcomb, 2010) and the need to replace lost roles and create a new sense of meaning in life through the process of meaning making (Kojola & Moen, 2016). Meaning making is considered a core process in adjusting to a broad range of stressful life situations such as illness, loss, and trauma (Park, 2008, 2013; Plattner & Meiring, 2006). The general meaning making framework (Park, 2010; Park & George, 2013) is based on the assumption that when encountering situations with the potential to challenge or stress their global meaning, individuals appraise the situations and assign meaning to them. Discrepancies between global and appraised situational meaning lead to distress, which can be reduced by meaning making efforts. These efforts include changing either the appraised or global meaning to reduce the discrepancy. Success leads to lower distress and better adjustment to stressful events.

Retirement adaptation can be seen as an activity in which searching for meaning and meaning making play a crucial role. Retirement is an opportunity for people to rethink their identity and find new meaning in their lives (Wang et al., 2014). Many previous studies have confirmed that when meaning making in old age is successful, it leads to better adaptation. The ability to find meaning in old age robustly correlates with psychological health and overall well-being (Hupkens et al., 2018; Reker & Wong, 2012; Steger et al., 2009). A stronger sense of meaning in life in old age predicts a reduction in depression (Reker, 1997), and in some older people meaning in life helps offset the potentially deleterious effects of negative life events on the symptoms of depression (Krause, 2007a). A high level of meaning in life is linked to better physical health too. Both Krause (2009) and Hill and Turiano (2014) found that older people with a high level of meaning in life had a lower probability of death during the study follow-up period than those with a lower level of meaning. Krause (2009) attributed this effect to the potentially important indirect influence of meaning operating through health.

We have argued that re-establishing meaning in life is an important component of the retirement adaptation process. However, this issue has rarely been addressed in the empirical literature. In their theoretical study, Froidevaux and Hirschi (2015) emphasize that work is no longer a source of meaning for retirees, but meaning at a broader level (that is, meaning in life) can still be found and/or achieved and maintained, through such sources as generativity, spirituality, and leisure activities. However, there is a lack of detailed empirical studies on meaning making in the retirement transition. The above-mentioned studies confirmed the importance of meaning in retirees, but specific studies focusing on how retirees make new or re-establish their meaning are not available. We believe that our study will provide deeper insights into this issue and it will be useful to those planning interventions aimed at successfully coping with retirement.

In our study, we decided to investigate meaning making in Slovak retirees who have recently transitioned into retirement. We adopted a qualitative approach—consensual qualitative research—because we wanted to obtain information from our participants without them having pre-given conceptions. In the interviews, we focused on questions such as meaning in life before and after retirement and changes in these. We also asked about experiences of meaning making during this process. Finally, we focused on the factors that help or hinder successful meaning making to help understood why some retirees are more successful than others at adaptation.

## Method

### Participants

Forty persons (75.5% of women) aged from 57 to 69 years *(M *= 63.36; *SD *= 2.47) participated in the study. They were retired from six months to four years (*M* = 2.8). They reported level of completed education: the majority had upper secondary education (n = 30; 75%), followed by a university education (9; 22.5%); and lower secondary schooling (1; 2.5%). Most were married (n = 27; 67.5%), or widowed (7; 17,5%), single (4; 10%), or divorced (2; 5%). Twenty-one participants lived in villages and 19 participants lived in a town/city.

### Procedure

To be eligible for this study, participants had to be retired and not working permanently, and to have been retired for a minimum of six months and a maximum of four years. The research was conducted in Slovakia and participants were recruited through institutes and associations for seniors and through the researchers’ personal networks. We used a snowball sampling technique. Before starting the interviews, participants read and agreed with the consent statement to participate in the research. Participants were fully informed about recording the interview, research aims, and conditions. They could terminate their participation at any time of the interview. The confidentiality of the participants was guaranteed, participants were informed that the data would be analysed together for the whole group, no names or other personal data would be collected, disclosed or published. All procedures performed in this study were in accordance with the ethical standards and with the 1964 Helsinki declaration and its later amendments or comparable ethical standards. Participants could choose the location of the interviews, most of them (*n* = 37) choose their homes, some of them (*n* = 3) a quiet café.

The semi-structured interviews were performed in person by the authors, who have previous experience of conducting qualitative research interviews. The questions concerned meaning making during the retirement transition as part of a broader interview focusing generally on retirement. The rest of the interview is not analysed in this study.

In line with consensual qualitative research methodology (Hill, 2012), a core set of following questions was formulated to achieve research goals: *What were the sources of meaning in your life before retiring? What were the sources of meaning in your life after retiring? How have you experienced a retirement transition in meaning in life? Has anything changed in this area, and if so, what has changed? What has been helpful to maintaining or recover your meaning in life after retiring? What, on the contrary, has been an obstacle for maintaining or recovering your meaning after retiring?* The average length of interview was 67 minutes, including a final debriefing conversation. The author of each individual interview removed all identifying data about the participants befora the data analysis. Then the interviews were transcribed by the second and third authors. During the transcription, any confidential information about the participants was deleted. Data were anonymized via the adoption of a code to identify each participant, and pseudonyms were used for qualitative quotes.

#### Data analysis

During the analysis, Consensual Qualitative Research-Modified (CQR-M; Spangler et al., 2012) was applied. In this method, the results expressed in domains and categories are built on ther base of the data without any theoretical pre-conceptions (Hill, 2012), and inner experiences and attitudes are subjected to an in-depth examination. During the CQR-M analysis, a team of judges separately code the data into domains, categories and subcategories in several steps. During each step, judges are talking over the results in order to achieve consensus on the final categorization (Hill, 2012). In our case, the team consisted of three authors of this study and one external auditor. CQR-M is recommended in situations, when samples are larger than typical number of participants in original Consensual Qualitative Research, and when data obtained are rather straightforward (Spangler et al., 2012).

First, the authors separately created a list of domains, based on their reading of the transcripts and in line with the previous literature and research questions. Then the list of domains was discussed until a consensus was reached. Consistent with the recommendations of Hill (2012) and based on the sample size (*N* = 40), the interviews were divided into four parts and analysed separately. Members of the research team separately analysed the transcriptions of the first ten interviews and sorted the data into categories and subcategories. Then, the team joined together and discussed the domain names, categories, and representative examples of the categories until a consensus was reached. Subsequently, the next tensomes of interviews were analysed one by one, and the coders evaluated how the new interviews saturated the categories. The saturation was attained after 30 interviews, as adding the last ten interviews did not bring any substantial changes in categories structure. In order to avoid the influence of groupthink of primary researchers, an external auditor (with the experience in CQR method) went through categories and subcategories and offered comments and suggestions (Spangler et al., 2012). Then the research team discussed and included suggestions of the auditor. When a final consensus was reached on the categories and subcategories, the data analysis was complete.

## Results

The first domain, *sources of meaning before retirement*, summarizes seven categories of pre-retirement sources of meaning our participants had (Table I).

**Table I.** Sources of meaning before retirement

Domain	Category	Subcategory	Representative item
**Sources of meaning before retirement**	**Family (28/70%)**	Maintaining family relationships (16/40%)	My family, first and foremost, my wife and my children, so that we are together and have common interests.
		To provide for family (8/20%)	It was certainly my family before retirement, to make sure that we were all well, healthy, that our children were happy, that we did not have to starve … we could live with the fact that we could go on vacation or buy what we wanted and needed. We didn’t have luxury, but we had enough to meet our needs.
		Family in general (7/18%)	And of course, my family.
		Taking care of children (5/13%)	To raise children, to marry them off, to take pleasure in them.
	**Work (24/60%)**	Self-realization (12/30%)	Work came first … I think the meaning of my life in the other third of my life was my job. My first priority … I wanted to achieve a lot of things in my job, as I am an ambitious person.
		Finances (5/13%)	Work hard and systematically, and thus make sure I had a good job and provide financially for the family.
		Acknowledgement (4/10%)	My job was always very important to me, I always tried my best and nothing less, therefore my boss liked me, too. It always pleased me when I was praised or when I received a reward, even a very small one, but I got it for a job well-done. I always went home really tired but feeling I was doing my job right rather than not working hard enough and going home feeling that time was just passing by.
		Work team (3/8%)	From the beginning work was definitely the meaning, we had a good work team, you learnt a lot, there was humour, laughter, we laughed together …
		Work in general (3/8%)	Work and of course those kids at work, that was my life.
		Work as a way to improve others‘ life (2/5%)	And work, of course. I thought that if I destroyed loads of ammunition, I would save so many humans lives. That was the meaning of my life, that I was doing something for other people this way.
	**Hobbies (11/28%)**		We spent the weekends at our cottage, I had a garden and I used to look after the flowers and plants …
	**Health (8/20%)**		So that we are all healthy, that is most important.
	**Spirituality (2/5%)**		I am also religious, so I go to church, I pray. I believe it fulfils me in some way and mostly it helps me. I was raised that way and I raised my children that way, so that also plays a huge role in my life.
	**Keeping calm interpersonal relations (1/3%)**		In general, I am satisfied with my life, I had no big problems with anybody, I used to live in peace even at my job, I never got involved in big conflicts with anybody.
	**Plans for retirement (1/3%)**		So, my goal before retirement was … that when I finally retired, I would do what I planned at home, what I had always wanted to finish. Living in the village is like, there is always something to do, something to work on, to improve … I looked forward to it, to doing the things I like to do.

For 70% of the participants, the main source of meaning before retirement was family. Participants described how important it was for them to maintain family relationships, to spend time with their partner and children. Specifically, women talked about the importance of giving children a proper upbringing and then seeing them marry, and men talked about the priority of providing financially for the family.

The second most common category was work as a source of meaning. For many participants, work was a form of self-realization; it was important for them to achieve goals and be successful. Moreover, some participants talked about the importance of being acknowledged in their job, for example, as a form of reward from their boss. Working hard was a way in which our participants could ensure they had a good job and were able to provide financially for the family. For three participants, having a good work team was especially important, not only to ensure good teamwork at work, but also for sharing positive experiences. For two participants, their work gave them meaning because they wanted to improve the life of others through it.

For 28% of our participants, meaning was derived from having meaningful hobbies, such as playing sport, reading books or looking after flowers and plants. For one fifth of retirees, the most important source of meaning was keeping healthy and the health of family members. A few participants valued the beneficial role of spirituality in life, maintaining peaceful interpersonal relationships and having retirement plans.

The second domain, *sources of meaning after retiring*, included nine categories (Table II). The most common source of meaning after retiring was the family. It was still important for our participants to maintain relationships with family members and to care for others, but looking after grandchildren became especially important. Work became less relevant and was no longer connected to employment, but mostly consisted of work around the house and in the garden. One participant described the importance of having a temping job and another described his desire to pass on the knowledge he had accumulated over his life onto the next generations by teaching at secondary school.

**Table II.** Sources of meaning after being retired

Domain	Category	Subcategory	Representative item
**Sources of meaning after retiring**	**Family (28/70%)**	Maintaining family relationships (15/38%)	Family. My sister, her husband, and extended family. We always try to help each other. We always visit each other, so that our relationship doesn’t fade.
		Taking care of grandchildren (12/30%)	It’s still my family … but now it’s more about taking care of my grandchildren, teaching them many new things, now it’s my way of giving to others.
		Caring for others (9/23%)	My husband is at home, after his stroke I had to adjust to everything that was new. So now all my activity is centred around him, cooking for him, taking him to the bathroom, turning him, adjusting his hand so he is comfortable, everything … it’s my main activity now.
	**Work (18/45%)**	Household, garden 10/25%	So, the meaning is still in work, but now it’s working at home, it still drives me to do things around the house, for the household, so I would say, before retirement the meaning was in needing to go to work, now you don’t have to work, but you still work.
		Temping job 1/3%	Now my temping job fulfils me, that I can work on something for four months, especially the opportunity to meet with other people connected to it.
		Generativity 1/3%	Passing on your knowledge and experience … I would like to teach at the secondary school if possible. I no longer want to work, on a full-time or part-time basis, but just teach one subject, that would make me happy caring for the young, upcoming generation, and that way capitalizing on my experience. Just as there is a full stop at the end of a sentence, this would be the natural result of my professional working life.
	**Keeping healthy (9/23%)**		To stay healthy, for myself and for my partner. That’s the most important thing now./That I wake up in the morning and see my husband lying by my side, that he is healthy, that he has not died. In the night I listen to see if he is breathing . That is the most important thing now, our health.
	**Freedom (4/10%)**		Now the meaning of life is that I am satisfied, that I can do what comes to mind, that I can go wherever I want, meet with people, take a rest … . The children are living on their own so nobody’s limiting me, now I decide what I want and what I don’t want, generally I feel I have greater freedom.
	**Spirituality (4/10%)**		The most important thing is god for sure. God is the meaning of my life. Faith comes first because my husband could die, the children may leave, but God always stays.
	**Social relationships (3/8%)**		Most importantly being able to meet with other people … I look for ways of meeting with people …
	**Rest (1/3%)**		You rest more than when you went to work.
	**Enjoying life** **(1/3%)**		To enjoy life while I can. Not worrying about what has happened, enjoying every single day when I wake up and I am healthy.
	**A good death** **(1/3%)**		Well, the meaning of being an old man is to die quickly. So, you don’t have to be taken care of, so you don’t have to go into a retirement home where you use diapers and stuff. This is my philosophy or my desire.

Keeping healthy was especially important for about a fourth of the participants. Some women described how important it was for them to wake up and see that their husband was still alive and breathing, and that they were relatively healthy. For 10% of participants, freedom became an important source of meaning after retiring. They enjoyed having the opportunity to do whatever they wanted whenever they wanted, and not being limited by work, with some having had to work during the weekend and on public holidays. Four participants described the importance of spirituality in their life, how they needed to find strength in their faith and how, through faith, they upheld the most important values in their life. One participant described the desire to enjoy life every day while he could and another talked about the importance of a good death, meaning a sudden death and not having to be looked after.

When performing the data analysis, we created a domain called *meaning making process*, describing whether something had changed in the meaning of life after retiring or, on the contrary, having to change or actively reconstruct their sources of meaning after retiring (Table III). Most of the participants had actively reconstructed meaning in life. Some participants explained that they drew on their previous meaning. For example, one participant said that before retirement meaning in life was about working and after retiring it was still about working, but it was a different type of work. Before retirement, he had a meaningful job which he liked and he knew that he needed to prepare for retirement by engaging in other meaningful activities so he could maintain this meaning. Therefore, he built a workroom where he created things out of wood and that gave him meaning. Other participants described finding it very difficult to stop working and so were trying to compensate for this loss by finding new activities or by setting new goals in life.

**Table III.** Meaning-making process during retirement

Domain	Category	Subcategory	Representative item
**Meaning making process**	**Active reconstruction of meaning** **(16/40%)**	Drawing on previous meaning (10/25%)	It was work before I retired and it is work after retiring, I just do what I really want to do now … I used to do a job I liked and enjoyed. My job was not monotonous like some people’s, doing the same thing every day. It was different for me because I was working in research. And now I also like my “job”, but I work in my own workroom. So, my day is fulfilled, I enjoy this type of work. I’m forever making something, even now for my friend’s birthday I’m making a present for him in my workroom. Then I’ll think of something new again, I’ll try, I’ll fail, I’ll improve something … . As I said, I don’t go to work, but I have enough work at home. I always find something to do.
		Trying to compensate for the loss of work (4/10%)	Even though I really miss the kids I used to teach in kindergarten, now I have my grandchildren and I can sometimes go to the kindergarten to visit and spend some time there, that is some compensation.
		Definition of new goals and plans (3/8%)	I had to think about the areas I wanted to get involved in after retiring. That was very important for me to work for myself finally. Because I think that for 43 years I worked for someone else, it was not always my choice, even though I liked my job, there were some restrictions and a strict framework … now, the strict framework has disappeared and is too wide. But I don’t want to live a life that is so, unplanned … because when the strict frameworks are missing, you can get lost in that.
	**Change of meaning (10/25%)**	More time for previous meaning (8/20%)	Family, hobbies, activities, nothing has changed in that, I still have the same activities now I am retired, the same hobbies, but I have more time for them.
		Disruption of meaning (2/5%)	It was all good when I was going to work, it was good when my husband was healthy, and we would have been looking forward to spending retirement together … he had a stroke … . It should have been better for us, it is much worse now …
	**Ongoing meaning 8/20%**		Nothing in my meaning has changed, as I said family came first before and it comes first now …

A quarter of participants described a change in meaning. Most of them had more time for their previous meaning, for example, family or hobbies still gave most meaning in life, but after retiring they had more time to spend on these sources of meaning. Two participants specifically experienced a disruption in meaning. They had experienced the death or serious illness of their partner and their sense of meaning in life had been completely shattered by the experience. In the last category, 20% of participants said that their sense of meaning in life was the same even after the transitioning to retirement, for example, it remained the family both before and after retirement, so their sources of meaning had not changed.

The fourth domain summarizes *factors that have helped seniors to maintain or recover meaning during their transition to retiremen*t (Table IV and V). We identified ten categories within this domain. Sixty-five percent of participants reported various protective attitudes that helped them to maintain or recover meaning during their transition to retirement. Most of the participants talked about the importance of being optimistic and having a proactive attitude, for example, performing activities which help keep the mind alert. Having insight and self-reflecting helped retirees to see that there are many people with problems and to be grateful for what they have in life. Also, being humble helped them to appreciate the little things in life and to not have overly high expectations. Fifteen percent of participants described how accepting the finality of life and thinking about death more often helped them to live a more meaningful life. For some participants, having the responsibility of caring for others and trusting in their abilities helped them maintain meaning in life.

**Table IV.** Beneficial factors for maintaining meaning of life in retirement

Domain	Category	Subcategory	Representative item
***H*elpful factors for maintaining the meaning of life**	**Protective attitudes (26/65%)**	Optimism and a proactive attitude 12/30%	I still have to keep my mind alert. Because if I only sat and did nothing, that would be worse. So, I still keep myself active and that is good for me, I don’t even know where the day goes./Always have a smile on your face, try to see everything positively … so that is it, positive thinking and enjoying little things.
		Insight and self-reflection (7/18%)	When you see how many people have problems, you value more that there is nothing serious to worry about. So, I enjoy these moments, when I have no pain, no big problems.
		Accepting the finality of life (6/15%)	After turning 50, I started to think more about death … I would definitely like to live, I like life, I want to live, but I accepted this situation. I think about death often and I think we should think about death every day. Because then we lead a different, better life
		Humility (4/10%)	I don’t have such high demands, I just want to live a decent life, not that type of thinking, like: “he has a nice car, I have to have one like that as well”. I have never wanted such things, I could have a car, but I don’t need it to be happy … you should be modest, you know, don’t take out loans, but use what you have and enjoy retirement.
		Responsibility (2/5%)	I have always felt some kind of responsibility for others, for myself, that I should take care of others—and not only during my working years, but also now that I am retired. So, having a responsible attitude is important.
		Gratitude (2/5%)	I am grateful for what I have in life, there are many people who can’t walk, don’t have anything to eat or drink, we also have relatively healthy air here …
		Trust in one’s abilities (1/3%)	People should trust in their abilities a bit, it’s really not over when you’re in your sixties and you can still do a lot of things.
	**Social support of meaning (26/65%)**	Sharing meaningful activities with loved ones (9/ 23%)	Since both me and my wife are retired, we work together in our garden, spend time together. So, if I was to say what was helpful to me, it’s my wife and the fact that we are still together, that we have been going through this transition and retirement together.
		Availability of care options for others (9/23%)	My family helped me for sure, gave me the opportunity to care for someone, that I have to take care of them, I can’t just leave everything be.
		Maintaining contacts (8/20%)	It’s not difficult because I have a big family, so we meet up with each other more. Now I live with my sister, so it’s different from living alone, you’d be completely alone …
		Accepting support from family and other people (6/15%)	Family, certainly a stable family background, peace, it has helped me the most, support from the man I have … When you know that there is comfort and peace waiting for you at home, you look forward to going home.
	**Availability of meaningful activities (9/23%)**		I like to be on the move, there is always something to do, I go out on my bike, to the garden, I am still active and that is so relaxing for me. I often wake up early and do many things, so at the end of the day, I look back and am very satisfied.
	**Health (8/20%)**		Again being health in retirement helped me mainly. I’m not an addict, I’m physically and mentally fit and my wife is the same. This makes me happy and encourages me. I want to keep it this way, because it is so encouraging.
	**Keeping the same goals 4/10%**		I always have to look for something to do. I find that with increasing age I forget things, my short-term memory is getting worse. There’s only one rule, solving crossword puzzles, learning English. I have to find some exercises on the Internet … Looking for things is important for me, so I don’t live a boring routine, but am trying to find new things to do.
	**Work availability (3/8%)**		There is still some work to do. Work in the garden, there is always work, from spring to autumn.
	**More freedom (3/8%)**		When I was working, I couldn’t just decide to go anywhere I wanted, I worked even at the weekend, or on New Year’s Eve … I have more free time now, that has changed. I can do what I want or go where I want and nothing keeps me from that.
	**Spirituality (3/8%)**		Faith in God, I try to live in accordance with the commandments, to do no harm to people and to be nice to people, and laugh more than curse.
	**Previous hobbies (2/5%)**		I used to read a lot when I was young, which has also helped me in retirement.
	**Later transition to retirement (1/ 3%)**		Of course, if I had retired as planned, at the age of 55, probably I would look back and think, what can I do? But I worked for ten more years so it went smoothly, it happened as it was supposed to happen.

**Table V.** Risk factors for maintaining meaning of life in retirement

domain	category	subcategory	representative item
**Risk factors for maintaining meaning of life**	**Lack of finances (5/ 13%)**		The limiting factor is lack of finances.
	**Loss of a loved one (4/10%)**		The loss of my wife, that was very hard on me.
	**Deteriorating health (3/8%)**		If only I was healthy … that’s the only thing limiting me, that I can’t get around so well, that my hip hurts, but otherwise I can’t complain very much.
	**Illness of a partner (2/5%)**		The risk factor was probably that I had to take care of my husband, that I couldn’t socialize so easily or have time to take care of myself.
	**Lack of activities, boredom (1/3%)**		It is just feels a bit different to how it did before, you get bored, especially during winter—you can’t work outside for such long hours. During the summer, you can always be outside and doing something …

Equally 65% of participants spoke about the importance of having social support. Specifically, that it is important to share meaningful activities with loved ones and to maintain contact with family members and friends even after retiring. Having various care options available to them was also important as was being able to accept support from family and people around them when needed.

About one fifth of participants said they could lead a meaningful life in retirement if they were in good health and could enjoy meaningful activities. For some, it was especially important to continue having goals in life, such as learning a foreign language so they could keep their mind and short-term memory active. For three participants, having greater freedom in making decisions about their free time was associated with a stronger sense of meaning of life in retirement. Spirituality also proved beneficial to three participants in maintaining or recovering meaning in life.

For 13% of seniors a lack of finances was a limiting factor in maintaining meaning in life, affecting, for example, their pursuit of hobbies. Four participants described the loss of a loved one, especially the death of a partner during the transition to retirement, as a risk factor in maintaining meaning in life, and in relation to feelings of grief and isolation. Two retirees explained that their husband was seriously ill and they had to devote all their time and energy to caring for him. This event not only led to many negative changes in life, but they also had no time or energy left for other sources of meaning, such as socializing or self-care. One participant experienced boredom in relation to retirement.

Based on the data analysis, we have identified *three typical cases* of meaning making. The most common case is individuals whose main source of meaning before retirement was the family and their job as a means of self-realization and a source of income. After retiring, their most important source of meaning in life remained the family, but more in relation to caring for grandchildren. Work became less important and mainly related to work around the house or garden, and associated with choice and not working out of obligation. Retirees, in general, have more opportunity to pursue their previous meaning in life, such as maintaining family relationships or engaging in hobbies. Freedom and staying healthy become increasingly relevant sources of meaning.

The second case is individuals whose job was their main source of meaning. Therefore, these retirees have to find a way of compensating for stopping work, by taking up a temping job or finding new hobbies for example. Trying to stay optimistic and proactive, and humility and gratitude help the meaning making process.

The last case is individuals whose main source of meaning before retirement was the family and who had planned to spend more time with their loved ones during retirement. After retiring, their meaning in life was disrupted by an unexpected, negative event—such as an illness or death of a partner. Accepting social support from others helps such individuals adapt to the new life situation.

## Discussion

The results of our research indicate several important aspects of meaning making during retirement. First of all, there was a close correspondence between sources of meaning in life before and after retirement. As mentioned above, employment and work can be a powerful source of meaning (Moen, 1996; Steger & Dik, 2010) and this was evident in our data (work and family were the two most significant meaning sources before retirement). Although there was a (rather small) decline in work as a source of meaning, it still remained one of the most important ways of deriving meaning in retirement. Family was another important source of meaning before and after retirement. Our results indicate that retirees tended to see retirement as a continuation of certain sources of meaning, rather than a disruption in life. Our results support the idea that retirement is a process as understood in the continuity theory of ageing (Atchley, 1989). Here retirement is not considered to be a demanding interruption but rather a chance to maintain a certain lifestyle (Quick & Moen, 1998; Von Bonsdorff et al., 2009). Von Bonsdorff et al. (2009) emphasized that in continuity theory these are individuals who were deeply engaged in their work and who are therefore able to maintain their daily routines by taking part in other valuable work activities. In our case, these activities included mainly housework and gardening or a temping job. However, these work activities do not only provide a daily routine and a means of filling the day, but, as our participants stated, may also provide them with a broader sense of meaning and passion.

Another important source of meaning playing an important role in life before and after retirement was the family. Social relationships occupy a substantial place in people’s meaning systems and are one of the most frequent sources of meaning throughout life (Debats et al., 1995; Krause, 2007b). Our results suggest that the family was the most crucial social source of meaning which accords with previous findings from research on people in different developmental stages (Lambert et al., 2009). Meaningful family relationships were not only mentioned frequently as sources of meaning, but were often reported as factors helping them maintain meaning. Sharing meaningful activities with a close person helps them experience meaning in these activities, as one of our participants stated in relation to gardening (see the example in Table IV). Krause (Krause, 2007a) emphasizes that because there are no clear standards for reaching meaning in life, the sense of meaning must be validated by another person. This can be achieved through feedback and guidance from a trustworthy person who may share his or her own experiences of meaning and provide examples and facilitation for older person’s own meaning.

However, our results also revealed differences in perceptions of the family as a source of meaning before and after retirement. Several participants mentioned that looking after the family (especially financially and healthwise) was a source of meaning before but not after retirement. Previous studies showed that the family remains an important source of meaning throughout life, but the conception of meaning in life depends on their personal role in the family (Fave et al., 2013). Our data indicate that retirement can bring about a change in the way the family serves as a source of meaning, from being responsible for providing financial security to maintaining family relationships between family members living in different places.

Taking care of family members is another important source of meaning. Although this can include both children and adults with special needs, taking care of grandchildren had special value for our participants. Participants who had grandchildren repeatedly mentioned them as an important source of meaning, which is in line with previous research confirming that grandparents experienced greater meaningfulness when they engaged in activities with their grandchildren compared to spending time alone or with other people (Dunifon et al., 2020). For newly retired persons, grandparenting can be both a strong source of meaning and satisfying compensation for a weaker sense of meaning in life associated with diminishing sources of meaning after retirement. We believe that grandparenting represents a form of generativity for retirees, which is considered to be a part of successful ageing and source of meaning (Hofer et al., 2014). The importance of generativity for retirees’ meaning is evident also from other areas, such as academic settings (Miranda-Chan & Nakamura, 2016). However, academic settings are specific, because generativity through mentoring can be achieved through emeritus positions. For most retirees whose work life comes to a more sudden end, family and grandchildren seem to be the most available source of generativity-related activities.

The results for the Meaning Making domain during retirement indicate that the retirement transition frequently involves the active reconstruction of meaning. Although some of the participants did not report a change in meaning, most revealed they had to put some energy into actively reconstructing meaning. Naturally, some of them drew on their previous meaning and continued to work but in other type of work. This result is in agreement with continuity theory (Atchley, 1989), which holds that retired people preserve their existing structures and adapt them to ageing-related changes. However, this is possible only if the retiree has access to the required resources (Wang et al., 2011), and for our participants this was mainly gardening and housework. On the other hand, retirees who were not able to access similar resources felt the need to compensate for the loss of work. Our results indicate that accessible resources play the most important role in retirement meaning making. As far as the general meaning making framework is concerned (Park, 2010; Park & George, 2013), retirees tried to maintain congruence between global and situational meaning by managing sources. If, for example, work was part of the retiree’s global meaning, a successful adaptation depended on the availability of work resources. Where resources are available, the retiree’s global and situational meaning is congruent and adaptation is successful. If new life after retirement does not provide opportunities to fulfill this value, the retiree has to reconstruct the situational meaning and find new resources. Our participants tried to do this by maintaining contact with pre-retirement work (e.g., visiting a kindergarten, see the example in Table III) and planning new activities for example. If they were successful, and the global and situational meanings became congruent, adaptation succeeded. Some of our participants did not experience successful adaptation because existing barriers (e.g., the serious illness of a spouse) did not allow them to access the relevant resources and so congruence was not achieved.

The two most important factors which helped retirees to maintain meaning are protective attitudes and social support of meaning. The fact that positive attitudes are positively related to meaning in life, and facilitate its intensity, has been confirmed in previous research. Meaning in life was found to be related to optimism (Ho et al., 2010), gratitude (Disabato et al., 2017), death acceptance (Tomer, 2013), and so on. Positive attitudes are especially important for dealing with meaning in adverse situations, such as illness and bereavement (Applebaum et al., 2014). As we described in the introduction, retirement can be accompanied by a sense of loss and distress related to the change in work status, but also by other negative aspects of older age, such as declining health. Positive attitudes can play an important role in adapting to these changes and maintaining satisfactory levels of meaning for several reasons. First of all, they enhance positive affect and emotional states (e.g., Trudel-Fitzgerald et al., 2019; Watkins et al., 2003). Also, positive attitudes can bring about a positive reframing or benefit, defined as the process in which the person reframes as positive something that was previously viewed as negative, for example, seeing it as an opportunity, the chance to learn something new, the chance to gain a new skill, and so on (Lambert et al., 2009). Through this process, positive reframing can lead to negative events being perceived as more meaningful.

Concerning social support of meaning, our results are in line with past studies which found that social interactions are an inevitable part of the meaning making experience. Debats, Dorst, and Hansen (Debats et al., 1995) revealed that experiences of meaningfulness in life were often linked to social interactions. These interactions involved family members, romantic partners, acquitances, and even unknown people. Krause (2007b) revealed that higher social support is associated with a stronger level of meaning over time. In their recent study, Krause and Rainville (2020) found that discovering of meaning was facilitated by the supportive social networks maintaining by older people. Participants in our research showed that this facilitation applies especially when sharing meaningful activities with a close person, maintaining contacts with close and wider family and friends, and in both providing and accepting care from others. Through these social interactions, retirees not only directly derive meaning in life, but also facilitate a stronger sense of meaning from sources which are not social in nature (e.g., some hobbies).

The most frequent risk factor mentioned by the retirees was lack of finances. Although some research has indicated there is no relationship between income and meaning in life (e.g., Sherman et al., 2011), later studies found a positive relationship between these two variables (Hill et al., 2016; Ward & King, 2016). We think lack of finances can have an impact on meaning as it causes negative emotions and is limiting, which may reduce the fulfilment of other values (e.g., restricting the number of hobbies owing to a lack of finances). We believe that lack of money leads to other resources becoming less accessible (see resource theory, Wang et al., 2011). Other risk factors reported by our participants related to a reduction in meaning resources. The loss of a close person can lead to feelings of loneliness and lack of opportunity to share meaning with other people. Declining health in the retiree and/or the person close to them presents an obstacle to realizing fulfiling and meaningful activities. Good health can contribute to experiencing meaning in life because healthier people lead more active, less burdensome, and more controllable lives (Czekierda et al., 2017). By contrast, poor health can contribute to a perception that life and the world are difficult, uncontrollable, and therefore less meaningful.

### Limitations

When interpreting the results of our study, it is important to take into account some limitations. At first, we decided to use a qualitative methodology to obtain a rich, contextualized understanding of meaning making during retirement adaptation, but while the results can be analytically generalized they cannot be statistically generalized (Polit & Beck, 2010). The second limitation concerns the retrospective nature of the research. The participants described current experiences but focused mainly on past experiences, which may have been subject to memory bias relating to a changed perspective or current mood (Smallwood & O’Connor, 2011). The final limitation deals with the sample’s education level. Most participants did not have a university degree, so the way they described their retirement experiences may differ from those with higher education.

## Conclusion

Many previous studies emphasized the importance of meaning in life in the retirement adaptation, however, there is a lack of detailed empirical studies on this topic. Our study fills this gap, and brings a better understanding of the meaning making process during retirement adaptation. The results show that this process is usually experienced as a continuation of retirees’ meaning sources, rather than as a serious disruption in their life period and a time of meaninglessness. However, some retirees may need to make an effort to adapt to a new situation, including finding or rediscovering new ways of meaning fulfilment. Especially when the process of retirement adaptation is accompanied by other negative changes, restriction of meaning sources can lead to a lack of meaning in life. Our results provide significant information that some retirees can have difficulties to restore meaning during the retirement transition and therefore they would need an assistance or intervention. Also, the findings on typical meaning sources as well as the significance of social networks can inform the interventions aimed at improving people’s ability to cope with retirement. However, our research focused on general population of retirees, so our findings on meaning making in the situation of adversity can be limited. Further research with special focus on specific groups of retirees (e.g., widows or widowers, the indigent etc.) is needed in order to understand in a greater detail how retirees make their meaning in such difficult situations.
